# Individualizing mechanical ventilation: titration of driving pressure to pulmonary elastance through Young’s modulus in an acute respiratory distress syndrome animal model

**DOI:** 10.1186/s13054-022-04184-w

**Published:** 2022-10-18

**Authors:** Álvaro Mingote, Ramsés Marrero García, Martín Santos González, Raquel Castejón, Clara Salas Antón, Juan Antonio Vargas Nuñez, Javier García-Fernández

**Affiliations:** 1 Anaesthesia, Critical Care and Pain Unit, Puerta de Hierro Majadahonda Universitary Hospital, Majadahonda. c/Manuel de Falla, 1, 28222 Madrid, Spain; 2 Anaesthesia, Critical Care Department and Pain Unit, Clinic Hospital, Barcelona, Spain; 3 Medical and Surgical Research Unit, Puerta de Hierro Majadahonda Universitary Hospital, Madrid, Spain; 4 Internal Medicine Laboratory, Puerta de Hierro Majadahonda Universitary Hospital Research Institute, Madrid, Spain; 5 Pathology Unit, Puerta de Hierro Majadahonda Universitary Hospital, Madrid, Spain; 6 Internal Medicine Unit, Puerta de Hierro Majadahonda Universitary Hospital, Madrid, Spain; 7 Faculty of Medicine, Autonomous University of Madrid, Madrid, Spain

**Keywords:** Acute respiratory distress syndrome, Driving pressure, Elastic modulus, Mechanical ventilation, Pulmonary elastance, Ventilator induced lung injury

## Abstract

**Background:**

Mechanical ventilation increases the risk of lung injury (VILI). Some authors propose that the way to reduce VILI is to find the threshold of driving pressure below which VILI is minimized. In this study, we propose a method to titrate the driving pressure to pulmonary elastance in an acute respiratory distress syndrome model using Young’s modulus and its consequences on ventilatory-induced lung injury.

**Material and methods:**

20 Wistar Han male rats were used. After generating an acute respiratory distress syndrome, two groups were studied: (a) standard protective mechanical ventilation: 10 rats received 150 min of mechanical ventilation with driving pressure = 14 cm H_2_O, tidal volume < 6 mL/kg) and (b) individualized mechanical ventilation: 10 rats received 150 min of mechanical ventilation with an individualized driving pressure according to their Young’s modulus. In both groups, an individualized PEEP was programmed in the same manner. We analyzed the concentration of IL-6, TNF-α, and IL-1ß in BAL and the acute lung injury score in lung tissue postmortem.

**Results:**

Global driving pressure was different between the groups (14 vs 11 cm H2O, *p* = 0.03). The individualized mechanical ventilation group had lower concentrations in bronchoalveolar lavage of IL-6 (270 pg/mL vs 155 pg/mL, *p* = 0.02), TNF-α (292 pg/mL vs 139 pg/mL, *p* < 0.01) and IL-1ß (563 pg/mL vs 131 pg/mL, *p* = 0.05). They presented lower proportion of lymphocytes (96% vs 79%, *p* = 0.05) as well as lower lung injury score (6.0 points vs 2.0 points, *p* = 0.02).

**Conclusion:**

In our model, individualization of DP to pulmonary elastance through Young’s modulus decreases lung inflammation and structural lung injury without a significant impact on oxygenation.

**Supplementary Information:**

The online version contains supplementary material available at 10.1186/s13054-022-04184-w.

## Introduction

Acute respiratory distress syndrome (ARDS) can lead to respiratory compromise. It is characterized by severe hypoxemia and a decline in pulmonary compliance [1]. In 2012, ARDS severity was classified according to the Berlin definition as mild, moderate, and severe [2] depending on the PaO_2_/FIO_2_ ratio. As a support therapy, mechanical ventilation (MV) may increase lung injury as ventilator-induced lung injury (VILI), and there are different ways through which VILI may occur: barotrauma, volutrauma, atelectrauma and biotrauma [3]. Gattinoni et al. have combined some variables that may play a role in VILI (driving pressure (DP), tidal volume (TV), flow, respiratory rate (RR)) in a single variable: the mechanical power (MP) [4], which represents the energy transfer from the ventilator to the lung. Meanwhile, two new concepts have emerged: stress (the force necessary to distort a material) and strain (the distortion of the material regarding its original state) [5]. Several protocols which aim to promote “protective mechanical ventilation” have been published to avoid VILI. These protocols consisted of low TV, such as 6 mL/kg of ideal body weight, as well as decreased inspiratory pressures (inspiratory peak pressure < 30 cm H_2_O, driving pressure < 15 cm H_2_O) [6] because driving pressure has recently been targeted as a risk factor for mortality [7].

**Fig. 1:**
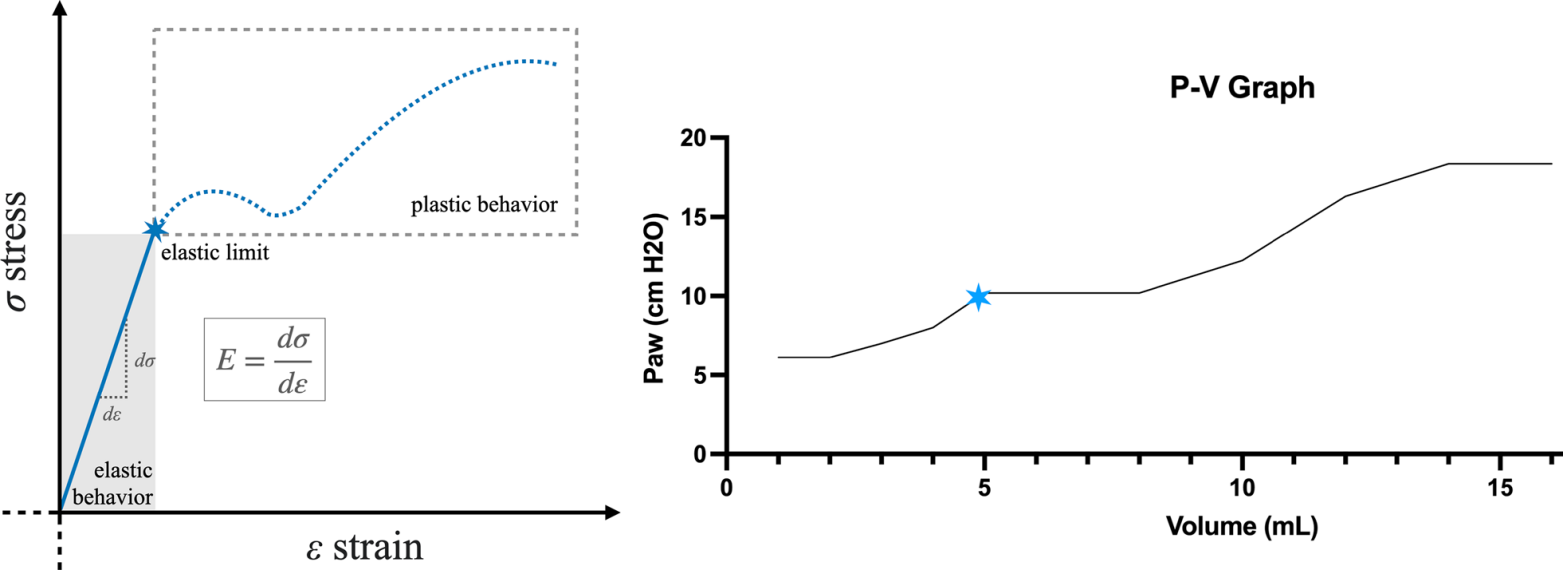
**A**: Young’s module: stress vs strain curve. **B**: Pressure volume curve obtained from one of the animals during our assessment of individualized DP. The blue star marks the elastic point

Despite the protective designation of these protocols, some studies showed that even these low TV (6 mL/kg) might involve the same stress and strain of 12 mL/kg in certain patients [8]. Recent clinical trials have highlighted that the decrease in ARDS mortality is not due to lower TV but to lung elastance and that we should focus on lowering DP rather than reducing TV [9, 10]. Tonnetti et al. proposed that one way to reduce VILI is to find the threshold of driving pressure below which VILI is minimized [10]. Understanding the lung as any material in nature, with its elastance and distortion capacity, opens a new avenues for lung study [11]. Young’s module studies the strain produced by specific stress in a material. Graphically (Fig. 1A), the pressure–volume curve initially shows a linear increase. Then, a flat zone shows the transition from an elastic to a plastic behavior, and the deflection in this zone indicates the maximum volume and pressure at which the material exhibits a total elastic behavior, that is, elastic limit. After this deflection, an exponential increase represents the plastic behavior in which elastic fibers are broken [12].

In this study, we propose a method to titrate DP to pulmonary elastance in an ARDS animal model using Young’s modulus and we aimed to study the effect of DP titration on VILI production (primary outcomes: lung inflammation and structural injury) as well as oxygenation. We hypothesized that optimization of DP would decrease VILI without a considerable impact on oxygenation.

## Materials and methods

### Ethical approval

The animals were handled according to the European and national regulations for the protection of experimental animals (2010/63/UE and RD 53/2013). Ethical approval (Ethical Committee CEEA/OH 002-001/2022 Ref. PROEX 034.2/22) was provided by the Institutional Animal Care and Use Committee of the Puerta de Hierro-Segovia de Arana Health Research Institute, Madrid, Spain. Our experiments were performed in accordance with the ARRIVE guidelines for experimentation in animal research.

### Experimental protocol

20 male Wistar Han rats aged 4–5 months were used. Their mean weight was 357 ± 21 g. An Additional file 1 provides a detailed experimental protocol. After the induction of general anesthesia and adequate monitoring (SpO_2_, temperature, invasive arterial blood pressure), we inserted a 16-gauge polyethylene catheter into the trachea. It was connected to an anesthesia Flow-i C20 (Getinge, Solna, Sweden) machine through a “Y connector” with a minimum dead space (< 0.2 mL) and a three-way stopcock (Additional file 1: Figure E1). Then we started MV as follows: pressure-controlled continuous mechanical ventilation (PC-CMV) with PEEP = 5 cm H_2_O, DP = 14 cm H_2_O, FiO_2_ = 0.5, sevoflurane 3% and RR = 40 bpm (adjusted to obtain an end-tidal CO2 between 40 and 45 mmHg). We established ARDS following the validated model proposed by Germann and Häffner [13] through repeated bronchoalveolar lavages (BAL). In our case, we performed 5 to 7 BAL with 10 mL/kg of warmed saline solution 0.9% to generate a severe ARDS according to the Berlin definition of ARDS [2]. After the BAL, we waited at least 15 min before obtaining blood gas samples with PC-CMV but with FiO2 = 0.8 to ensure post-ARDS oxygenation (SpO_2_ > 92%). The material obtained in the first two BALs was stored in a test tube and transported to the laboratory (we collected BAL prior to the ARDS generation to avoid the bias of describing an increased marker/population which would have been increased prior to ARDS generation).

### Individualized DP titration

After setting an individualized PEEP for each animal in the individualized DP group (a detailed protocol of experimentation is provided in an Additional file 1), we calculated for each animal their individualized DP (adjusted to pulmonary elastance). We changed the ventilatory mode to continuous positive airway pressure (CPAP) for the DP titration, with the individualized PEEP. We interrupted the connection between the anesthesia machine and the rat by closing the Flow-I port from the 3-way stopcock. Then we introduced a known volume of 14 mL of oxygen (in steps of 1 mL each step) through the stopcock, and we measured Pmean for each mL that we introduced. We introduced the data in the software Excel 16.59 and obtained a pressure–volume graph.

An example is presented in Fig. 1B. According to the Young’s curve, the maximum slope of the graph would represent the elastic limit, followed by the flat zone, which indicates the transition zone. We considered this point as the individualized driving pressure. It is important to emphasize that the first part of the graph (‘elastic behavior’) corresponds to a flat zone in the animal’s graph because the individualized PEEP already opens the lung.

### Experimental groups

A computer-generated random list selected one of the two groups. In the standard group, 10 animals received 120 min of MV with the standard ventilation parameters described previously (PEEP = individualized PEEP, FiO_2_ = 0.8, RR = 55 bpm, DP = 14 cm H_2_O always checking that TV was < 6 mL/kg). In the experimental group, 10 animals received MV for 120 min with the following parameters: PEEP = individualized PEEP, DP = individualized DP, FiO_2_ = 0.8, RR = 55 bpm). Hence, the total MV time was 150 min: 30 min during the induction and 120 min with the parameters we described. After this time, we finished the experiment, obtained arterial blood for blood gas analysis, and performed two BALs with the same solution and volume as before. The obtained material was kept in a test tube and transported to the laboratory. The animals were killed, and their lungs were collected (an Additional file 1 provides a detailed experimental protocol). In this Additional file 1: Tables E1 and E2 show detailed ventilatory parameters for each animal group, and Additional file 1: Figure E2 shows Young’s curve for each animal of the experimental group.

### BAL analysis

Additional details on the BAL processing can be found in Additional file 1. Samples were analyzed using a FACSort flow cytometer. We also measured the concentrations of tumor necrosis factor (TNF)-α, interleukin (IL)-6, and IL-1β using enzyme-linked immunosorbent assays (ELISAs) (Elabscience, China) according to the manufacturer's protocol.

### Histopathological grading of VILI

After processing the lungs (Additional file 1), two pathologists’ experts in interstitial pneumopathies blindly analyzed lung injury through the most used score to analyze VILI: the ALI score [14, 15]. This score has four items: (a) alveolar capillary congestion, (b) hemorrhage, (c) infiltration of neutrophils into the airspace or the vessel wall, and thickness of the alveolar wall, and (d) alveolar wall thickness/hyaline membrane formation. Every item received punctuation ranged from zero to four, corresponding zero to normal findings, one to mild (< 25% of the surface), two to moderate (25–50%), three severe (50–75%), and four to very intense (> 75%) lung involvement. The score used for the results was the mean score for each sample from both pathologists.

### Statistical analysis

Sample size and power calculations were based on previous data from similar studies and determined using the Granmo 7.12 software program (Institut Municipal d’Investigació Mèdica, Spain). Ten animals per group were deemed adequate to accept an alpha risk of 0.05 and a beta risk of 0.05 in a two-sided test. A normality test (Kolmogorov–Smirnov test with Dallar–Wilkinson–Llilliefor *p*-value) was performed to analyze quantitative variables. If the sample followed the normal distribution, quantitative data were shown as mean [95% interval confidence for the mean], and a *t* test was performed to check for differences between groups. If they did not, quantitative data are shown as median [p25, p75]. If the data distribution was parametric, a t test was performed to check for differences between groups, and a Mann–Whitney test was performed if it did not. Qualitative variables are presented as % [**p25, p75**], and after checking whether the collected variable met the minimum characteristics required, a *χ*^2^ test with Fisher correction was performed to check for differences between groups. Statistical analysis was performed using Prism Graphpad 9.0 software.

## Results

### ARDS

Both groups presented severe ARDS. Animals from the control group showed an initial PaO_2_/FiO_2_ ratio of 79 [68–87] versus 71 [62–79] (*p* = *0.33*) in the experimental group after the generation of ARDS. We observed an individualized PEEP of 12 cm H_2_O [10 cm H_2_O–12 cm H_2_O] in the control group versus 12 cm H_2_O [9 cm H_2_O–12 cm H_2_O] in the experimental group (*p* = *0.68*). The animals in the control group received PC-CMV with a median DP of 14 cm H_2_O [14 cm H_2_O–14 cm H_2_O] over the individualized PEEP, while the experimental group received PC-CMV with the individualized DP that we estimated for each animal (see Additional file 1 for details on DP for each animal of the group). Globally, they showed a median DP of 11 cm H_2_O [11 cm H_2_O–14 cm H_2_O] (*p* = *0.01*) over their individualized PEEP.

### BAL analysis

**Fig. 2:**
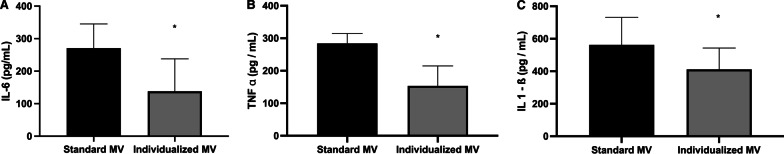
**A**. IL-6 concentration in BAL after 150 of MV (*p* = 0.02). **B**. TNF-α concentration in BAL after 150 of MV (*p* < 0.01). **C**. IL 1-ß concentration in BAL after 150 min of MV (*p* = 0.05)

Prior to ARDS, we did not detect significant concentrations of IL-6 nor TNF-α and the median concentration of IL-1ß was 372 pg/mL [313 pg/mL–483 pg/mL] in the control group versus 366 pg/mL [286 pg/mL–388 pg/mL] in the experimental group (*p* = 0.25). After 150 min of MV (Fig. 2A–C), the median concentration of IL-6 in the control group was 270 pg/mL [230 pg/mL–340 pg/mL] while in the experimental group was 155 pg/mL [42 pg/mL–210 pg/mL] (*p* = 0.02). The median concentration of TNF-α of the control group was 292 pg/mL [259 pg/mL–305 pg/mL] versus 139 pg/mL [110 pg/mL–217 pg/mL] in the experimental group (*p* < 0.01). Finally, the median concentration of IL-1ß in the control group was 563 pg/mL [376–732 pg/mL] versus 331 pg/mL [311–572 pg/mL] in the experimental group (*p* = 0.05).

### Blood gas

We did not find any statistical differences in the blood gas immediately after the development of the ARDS (*p* = 0.64). After receiving 150 min of MV, we observed a median PaO_2_ of 121 mmHg [109–143 mmHg] in the control group compared to 137 mmHg [91–181 mmHg] in the experimental group (*p* = *0.84)*. The median PaCO_2_ in the control group was 75 mmHg [59–90 mmHg] compared to 92 mmHg [68–102 mmHg] in the experimental group (*p* = *0.15*). The median lactate concentration was 1.8 mmol/l [1.0–3.7 mmol/L] in the control group versus 2.0 mmol/L [1.4–3.9 mmol/L] in the experimental group (*p* = *0.56)*.

### Cellular population

Prior to ARDS, in the control group, we found that 54% [24–90%] of the population were lymphocytes, compared to 43% [29–70%] in the experimental group (*p* = 0.77). In the control group, 4% [1–6%] were monocytes compared to 3% [2–5%] in the experimental group (*p* = *0.47*). We found that 41% [9–70%] of the cell population in the control group corresponded to polymorphonuclear leukocytes compared to 5% [28–65%] in the experimental group (*p* = 0.69). After 150 min of MV, we found that 96% [95–97%] of the cell population in the control group corresponded to lymphocytes, compared to 79% [65–90%] in the experimental group (*p* = 0.05). 2% [1–3%] corresponded to monocytes/macrophages in the control group compared to 3% [2–5%] in the experimental group (*p* = 0.1). Finally, we observed 2% [2, 3] of polymorphonuclear leukocytes in the control group compared with 13% [2–24%] in the experimental group (*p* = *0.05*).

### Histopathological grading of VILI

**Fig. 3:**
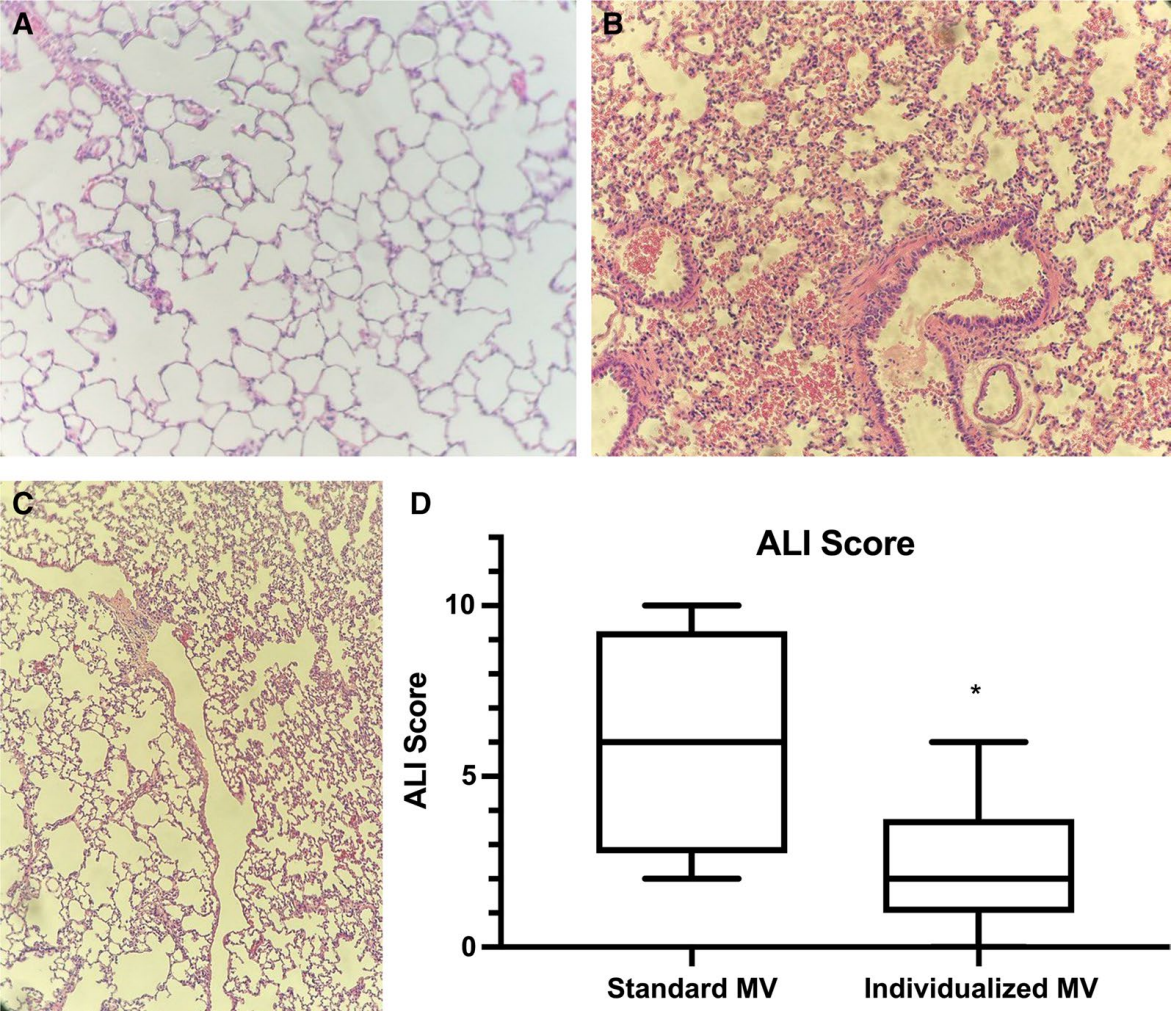
**A**. Postmortem histological slide showing upper right lobe of a rat from the experimental group, showing minimal capillary congestion. **B** and **C**. Postmortem histological slide showing upper right lobe of a rat from the control group showing considerable capillary congestion, active hemorrhage and peri-bronchial edema and increase of the thickness of the alveolar membrane, even alveoli rupture possibly due to barotrauma (**C**). **D**. Global ALI score for control and experimental groups (*p* = 0.02)

The control group showed a median global ALI score of 6.0 points [2.8 points–9.3 points] versus 2.0 points (1.0 points–3.8 points) in the experimental group (*p* = 0.02) (Fig. 3A–D). Regarding alveolar capillary congestion item, we found a median score of 2.0 points [1.0 points–3.0 points] in the control group versus 1.0 point [1.0 points–2.0 points] in the experimental group (*p* = 0.12). Regarding hemorrhage, we observed a median score of 0.0 points (0.0 points–3.0 points) in the control group versus 0.0 points (0.0 points–1.0 points) in the experimental group (*p* = *0.28*). Regarding the infiltration of neutrophils into the airspace or the vessel wall and the thickness of the alveolar wall, we observed a median score of 2.0 points (1.0 points–2.5 points) in the control group versus 1.0 points (0.0 points–1.0 points) in the experimental group (*p* = *0.03*). Finally, regarding wall thickness/hyaline membrane formation we observed a median score of 0.0 points (0.0 points–2.0 points) in the control group versus 0.0 points (0.0 points–1.0 points) in the experimental group (*p* = *0.28*).

## Discussion

Some authors consider that protective MV protocols are based on reducing the DP to less than 15 cm H_2_O to prevent VILI. We observed that titration of the DP to the maximum elastic point through Young’s modulus would diminish the inflammatory response associated with VILI and the consequent structural changes.

The onset of VILI has focused on the protocols and guides to apply a protective MV, but the term ‘protective MV’ is unclear as the exact parameters are in discussion. Complex concepts such as mechanical power, stress, strain, and special efforts to diminish DP have recently appeared [16]. Still, its application to our clinical practice is complicated partly due to the heterogeneity of the lung suffering ARDS. Although some authors have studied the possible impact of decreasing TV and DP according to pulmonary elastance [11], we did not find studies that described the individualization of tidal volume and driving pressure further than the consideration of ideal body weight or a specific limit for DP (< 15 cm H_2_O).

The application of our method to our severe ARDS model showed that the individualized DP differs from the traditional limits. With this decrease in DP, we did not observe significant reductions in oxygenation, although we observed a tendency to increase PaCO_2_. After more than two hours of MV, we have described that those who received individualized MV showed less lung inflammation, and a lower proportion of lymphocytes in BAL (although its role is to be established) as well as less lung injury (especially when it comes to alveolar capillary congestion and leukocyte migration).

Theoretically, according to Young’s module [12], an appliance of a DP higher than the individualized would not provide more elasticity to the lung, but, instead, it would start to break its elastic fibers. This event may promote lung inflammation and structural lesion. The calculus of the individualized DP allows us to determine the maximum DP that would deliver the maximum TV to be applied without worsening the established lung injury. Another benefit is that we would always know the top of DP to apply, and in case a patient would need higher levels of DP to maintain oxygenation or to maintain PaCO_2_ in a reasonable range, perhaps other therapies could be considered (ECMO, ECCO_2_R), as recent studies suggest [17, 18], to avoid severe hypercarbia while applying individualized protective ventilation according to the daily physical properties of the lung, but this should a matter of future studies. In addition, given that during ARDS, the lung may evolve from an inflammatory phenotype to a fibrotic form (especially in SARS-CoV-2 ARDS [19]), daily analysis of the individualized DP and elastic limit may characterize the lung phenotype, providing knowledge of the evolutive tendency of the lung injury. Many clinicians consider that limiting DP to less than 15 cm H_2_O is enough to protect the lungs, and these findings suggest that individualized DP according to the individual elastic point (if allowed by the clinical situation of the patient) might be a better message, although this should be confirmed in future studies in humans.

However, the present study has some limitations. First, as we are not studying the prognosis, we do not know the impact this method may have (although we infer that a reduction in inflammation and lung injury would imply a better prognosis) and should be studied in the future. Second, our ARDS model might not accurately represent every type of ARDS in humans, and the time that the animals received MV was lower than that of ARDS patients in our clinical practice. However, it was sufficient to observe changes in inflammation and lung injury and we chose this model because it has been validated for pre-clinical ARDS experimentation, while other models might involve an activation of inflammatory cascades that could potentially interfere with the inflammatory response that we aimed to study. In addition, in these experiments with our ARDS model, we only employed male rats (to homogenize basal characteristics of the sample and to minimize the impact of the gender of the animal on lung mechanics). In this sense, individualized PEEP was obtained through the super syringe method in the expiratory curve, which is not common in clinical practice. Moreover, although we have indeed worked with relatively low TV, the Flow-I machine is approved for neonates (the precision of the volume sensor is near 1 mL), and we preferred PC-CMV to VC-CMV to diminish potential erroneous insufflation of one or two extra mL. Although there are some considerations for our physical model (online supplementary data) and considering that we did not aim to calculate a specific value for pulmonary elastance, but it changes over the time with driving pressure, the actual P–V curves for each animal are indeed consistent with the theoretical reasoning exposed. Finally, although we had a relatively small sample size, we obtained sufficient statistical power to identify differences between the groups. Future studies in superior mammals and humans could address these concerns and establish the applicability of our method.

## Conclusion

In our experimental model of severe ARDS, we observed that adequation of DP to the elastic limit of the lung through Young’s modulus decreased lung inflammation and structural lung injury without a significant impact on oxygenation. Future studies on the applicability of this method to humans and the effect on the prognosis should be performed to determine its actual utility.

## Supplementary Information

**Additional file 1.** Details of the experimental protocol.

## Data Availability

Not applicable (Data from experiments are available from the corresponding author on reasonable request).
