# Supplementary material for: Individualizing mechanical ventilation: titration of driving pressure to pulmonary elastance through Young’s modulus in an acute respiratory distress syndrome animal model
Source: Crit Care. 2022 Oct 18;26:316. doi: 10.1186/s13054-022-04184-w (PMC9578179; doi:10.1186/s13054-022-04184-w)

***Individualizing mechanical ventilation*:**

***Titration of driving pressure to pulmonary elastance through Young’s module in an acute respiratory distress syndrome animal model*.**

*Álvaro Mingote,^1,6^, Ramsés Marrero García^2^, Martín Santos González^3^, Raquel Castejón Díaz^4^, Clara Salas Antón^5,6^, Juan Antonio Vargas Nuñez^4,6^, Javier García Fernández^1,6^.*

**Online Data Supplement**

***Suplementary material and methods***

**Animals and ethical approval**

The animals were allowed to acclimatize under veterinary supervision for at least one week before the experiments. Rats were housed in groups of 8 to 10 in U-TEMP polyetherimide cages, with bedding, environmental enrichment, and free access to food and water, and maintained on a 12-hour light/12-hour dark cycle under controlled environmental conditions (50-70% relative humidity, 20 ± 2 ^o^C temperature). Before the study, the health status of the animals was assessed, and any alteration was considered a reason for exclusion. Each experiment was performed in the morning (09:00-14:00 hours). On each study day, one unmedicated animal was transported to an operating room close to the housing room, where the animal was weighted. Before the beginning of the experiments, we routinely checked the condition of the anesthesia machine, as well as we calibrated pressure sensors and established a room temperature of 24 – 25 ºC.

**Experimental protocol**

General anesthesia was induced with Sevoflurane 8% vaporized in a mixture of air and oxygen with an inspired fraction (FiO2) of 0.5. After obtaining adequate anesthetic depth, we monitored the animal with a pulse oximeter (Infinity® Delta; Dräger, Lübeck, Germany) in the lower extremity to record hemoglobin oxygen saturation (SpO_2_). A peripheral 24-gauge polyethylene catheter was then introduced in one of the lateral caudal veins in the tail to get peripheral venous access. Then, 0.1 mg/kg of morphine chlorydrate was administrated.

After we checked the anesthetic depth again, we dissected the anterior cervical space to expose the trachea and the left carotid artery, isolated from the vague nerve. We inserted a 24-gauge polyethylene catheter in the left carotid artery, connected it to a pressure sensor, and fixed it to the subcutaneous tissue through silk sutures to continuously monitor arterial pressure. Then we inserted a 16-gauge polyethylene catheter in the trachea and fixed it to the trachea with silk sutures to avoid potential leaks.

Following endotracheal intubation, we administered 0.6 mg/kg of rocuronium, and the 16-G catheter was connected to an anesthesia Flow-i C20 (Getinge, Solna, Sweden) machine through a “Y connector” with a minimum dead space (< 0.2 mL) and a three way stopcock (figure E1). Then we started MV with the following basal parameters: PC – CMV with PEEP = 5 cm H_2_O, DP = 14 cm H_2_O (hence, inspiratory peak pressure 19 cm H_2_O), FiO_2_ = 0.5 Sevoflurane 3%, and RR = 40 bpm (these parameters were then adjusted if necessary to obtain an end-tidal CO_2_ between 40-45 mmHg). We also monitored body temperature through a rectal probe to maintain normothermia (37-39 ºC) with a hot air blanket (EQUATOR® Convective Warmer; Smiths Medical ASD, Minneapolis, USA). Once the general anesthesia was correctly induced, and after assessing adequate hemodynamic stability, we established the ARDS.

The ARDS was established following the validated model proposed by Germann and Häffner in 1998 [1]. To summarize, it is shown through repeated bronchoalveolar lavages (BAL). In our case, we performed 5 to 7 BAL with 10 mL/kg of warmed saline solution 0.9% to generate a severe ARDS according to the Berlin definition of ARDS [2]. After the BAL, we waited at least 10 minutes with the basal parameters of our PC – CMV but with FiO2 = 0.8 to ensure post-ARDS oxygenation (SpO_2_ > 92%). To verify the severity of the ARDS, we obtained a blood sample from the carotid artery and measured blood gas to check that the PaO_2_/FiO_2_ ratio was less than 100. If this criterion was not met, we repeated the BAL as often as needed to accomplish it. The material obtained in the first two BALs was kept in a test tube and transported to the Internal Medicine Lab, where it was processed as described below.

For the maintenance of anesthesia, sevoflurane at 2.5% was used, and we administered 0.3 mg/kg of rocuronium every 30 minutes. In addition, rats received 10mL/kg/h of normal saline 0.9%. If the mean arterial pressure was less than 60 mmHg, we administrated 10 mL/kg of saline solution (NS 0,9%). There were no differences in the amount of fluid therapy received between both groups. An extra bolus of morphine chlorydrate of 0.1 mg/kg was administrated if the heart rate was higher than 200 bpm.

**Individualized PEEP assessment**

In both groups, after 15 minutes from the ARDS, we calculated the individualized PEEP for each animal through the super syringe method: after a recruitment maneuver (with a constant DP of 15 cm H_2_O, increasing PEEP 5 cm H_2_O at each step of 5 respiratory cycles until reaching 20 cm H_2_O of PEEP), starting with PEEP of 0 cm H_2_O (to obtain the inspiratory pressure-volume curve) and closing the port from the Flow-I machine in the three-way stopcock, we introduced 6 mL/kg of oxygen through the three-way stopcock connected to the intra-tracheal catheter (to avoid dead space between the anesthesia machine and the catheter). Then we registered mean airway pressure (Pmean). Then we opened the port from the Flow-I machine and increased PEEP by one cmH_2_O for 10 respiratory cycles. During expiration, we closed the port from the Flow-I machine in the three way stopcock and introduced 6 mL/kg of oxygen, registering Pmean. We repeated this procedure until reaching PEEP of 20 cm H_2_O by increases in PEEP of 1 cm H_2_O, and we repeated the whole process but decreasing PEEP from 20 cm H_2_O in steps of 1 cm H_2_O and reaching to PEEP 0 cm H_2_O (to obtain the espiratory pressure-volume curve), registering Pmean for each reduction of PEEP. We introduced these data in the software Excel 16.59 for Mac to individualize PEEP (the PEEP with the best compliance in the espiratory pressure-volume curve). A new recruitment maneuver (as described previously) was performed, and PEEP was decreased to the level of PEEP that we obtained to be the individualized PEEP.

**Individualized DP titration**

After setting an individualized PEEP for each animal, in the individualized DP group, we calculated for each animal their individualized DP (DP adjusted to pulmonary elastance). We changed the ventilatory mode to CPAP with the individualized PEEP for the DP titration. We interrupted the connection between the anesthesia machine and the rat by closing the Flow - I port from the 3-way stopcock. Then we introduced a known volume of 14 mL of oxygen (separately in steps of 1mL each step) through the stopcock connected to the intratracheal catheter, and we measured Pmean for each mL that we introduced. We introduced the data in the software Excel 16.59 for Mac, and we obtained a pressure–volume graph. An example of one of the animal’s graph is presented in figure 1 – B and detailed curves from the experimental group are shown in figure E2. According to Young’s curve, the maximum slope of the graph would represent the elastic limit, followed by the flat zone that indicates the transition zone. Hence, we considered this point as the individualized driving pressure. It is important to emphasize that the first part of the theoretical graph (‘elastic behavior’) corresponds to a flat zone in the animal’s graph because the individualized PEEP already opens the lung.

**Physical considerations on the model**

**With this model we did not aim to calculate the elastance of the lung but to determine the elastic limit of the lung (the maximum theoretical stress that an elastoplastic material can withstand without undergoing permanent deformation) through the Young modulus. Gattinoni and Marini indeed described Young modulus (‘*specific lung elastance*’, SLE) as constant and linear [3]. However this can only be assumed if the material behaves as totally elastic and non as a plastic (no permanent deformation exists after the application of a force). With our model, we decided to calculate directly Young modulus assuming that lung’s behavior is nonlinear. According to Hooke’s law for non-linear material, we must study SLE by stress/strain regions, calculating their differential for a given moment (the Young curve showed). This curve shows a non-linear behavior in severe ARDS when a considerable force is applied to the lung. We had to assume the following:**

***Regarding stress***

1. **In a mechanically ventilated lung, deformation forces are represented by the air pressure exerted by the ventilator through the tube: we considered the pressures of the ventilator since the pressures in the system tend to balance and consequently these pressures should be the same inside the lung when performing a ventilatory pause.**
2. **In a mechanically ventilated lung, PEEP also generates lung distension. Given that we cannot calculate the distension generated by PEEP at a given moment, we considered that this distension under stable circumstances remain unchanged in interval times of 1 minute to 1 minute. Hence, we assumed that the force that deform the lung will be the transpulmonary pressures discounting the PEEP for a given and stable moment.**
3. **Given these assumptions, we employed the pressures exerted by the ventilator in a pressure controlled continuous mandatory ventilation. In this sense, we concluded that for a given moment, differences of tensile forces in the lung are given by the pressures in ventilation since the external forces (as well as PEEP) remain constant, at least in the measurement of our experiment (for a given moment).**

***Regarding strain:***

**Strain is described as the product of flow and functional residual capacity (FRC). As we explained previously, we aimed to calculate differentials and not the absolute value for a given time. We studied for each point a respiratory cicle, hence the ventilatory time would be constant. In this sense, it is necessary to assume that FRC is not modified during the period of our mesure, which is why we performed a recruitment maneuver before the experiment to avoid new areas to be opened during the experiment. Hence, stress variation for a given moment would be proportional to Vt.**

**Although there are some assumptions for our physical model and considering that we were not able to calculate a specific value for pulmonary elastance, but its change over the time with driving pressure, the actual P-V curves for each animal are indeed consistent with the theoretical reasoning exposed. It is well known that the first deflection in Young’s modulus curve obtained is related to the elastic limit where the lung begins to behave as a partial plastic material. This is consistent with our observations in the experiment (lower interleukyns related to VILI, lower structural damage).**

**Experimental groups**

A computer-generated random list (Microsoft Excel 2016) selected one of the two groups. In the Standard DP group 10 animals received 120 minutes of MV with the standard ventilation parameters described previously (PEEP = individualized PEEP, FiO_2_ = 0,8, RR = 55 bpm, DP = 14 cm H_2_O always checking that TV was < 6mL/kg). In experimental group, 10 animals received MV 120 minutes with the following parameters: PEEP = individualized PEEP, DP = individualized DP, FiO_2_ = 0.8, RR = 55 bpm) (table E1, E2). Hence, the total MV time was 150 minutes: 30 minutes during the induction and calculation of the parameters and 120 minutes with the parameters we described. After this time, we finished the experiment, we obtained a blood sample from the carotid artery for blood gas analysis, and we performed 2 BALs with the same solution and volume as the previous one, and the material obtained was kept in a test tube, and transported to the Lab. The animals were sacrificed by increasing inspired Sevoflurane to 8% and through the administration of 150 mg/kg of sodic pentobarbital. Once asystole was established, we dissected the thorax, collected the lungs, and introduced them into a tube with 10% formaldehyde.

**BAL analysis**

***BAL Processing***

Bronchoalveolar lavage fluid (BALF) samples, kept cold, were processed in the following four h as follows: first, the BALF was filtered through a 70-mm cell strainer (BD Biosciences, USA) to remove mucus; then, the cellular content in the BALF was recovered by centrifugation at 300g for 10 min. The supernatant was collected and stored at -80°C for further analysis (ELISA). The cell pellet was resuspended in 2 mL of RPMI-1640 (Bio-Whittaker Europe, Belgium) to analyze the cellular influx in the lungs.

***Analysis of cellular subsets in the BALF by Flow Cytometry***

BALF cells were stained for 30 min at four °C with an anti-rat CD11b/CD11c monoclonal antibody coupled to fluorescein isothiocyanate (FITC) (clone REA325, Miltenyi Biotec, Germany). Anti-rat IgG irrelevant antibody was used as a negative control of staining. Then samples were washed in PBS to remove unbound antibodies and immediately analyzed in a FACSort flow cytometer with the CellQuest and the Paint-a-Gate Pro software (BD Biosciences, USA).

The staining and forward scatter component (FSC), and side scatters component (SSC) criteria were used to analyze leukocyte populations. Lymphocytes were identified using forward, side scatter as CD11b/CD11c negative cells, neutrophils were the CD11b/CD11clow population, and macrophages were identified as CD11b/CD11c^high^ cells.

***Measurements of inflammatory cytokines in BALF***

Concentrations of tumor necrosis factor (TNF)-α, interleukin (IL)-6, and IL-1β were detected with enzyme-linked immunosorbent assays (ELISAs) (Elabscience, China) according to the manufacturer's protocol. Briefly, standards or samples were added to each micro-ELISA plate well that were pre-coated with an antibody specific to the corresponding cytokine and combined with the specific antibody. Then a biotinylated detection antibody specific for each cytokine and Avidin-Horseradish Peroxidase (HRP) conjugate were added successively to each micro plate well and incubated. After washing away free components, the substrate solution was added to each well. Wells containing any of the cytokines, biotinylated detection antibody, and Avidin-HRP conjugate appeared in color. The optical density (OD) was measured spectrophotometrically at a wavelength of 450 nm. The OD value is proportional to the concentration of cytokines, therefore the concentration of cytokines in the samples could be calculated by comparing the OD of the samples to the standard curve. Detection range was 15.63-1000 pg/mL for TNFα, 12.5-800 pg/mL for IL-6 and 31.25-2000 pg/mL for IL-1β.

**Histopathological grading of VILI**

**For histological analysis, lungs were removed from post-mortem animals and fixed in a 10% formaldehyde solution. Then they were transported to the hospital's Pathology Laboratory, where they were embedded in paraffin and 4-μm-thick samples of the right upper lobe were sectioned using a rotary microtome (HM 355S; Thermo Fisher Scientific, Waltham, MA, USA). Afterwards, the paraffin was removed and they were dehydrated to stain them using the hematoxylin and eosin staining technique.**

After processing the lungs two pathologists experts in interstitial pneumopathies blindly analyzed lung injury through the most used score to analyze VILI: the ALI score [4 – 8]. This score has four items: a) alveolar capillary congestion, b) hemorrhage, c) inﬁltration of neutrophils into the airspace or the vessel wall and thickness of the alveolar wall, and d) alveolar wall thickness/hyaline membrane formation. Every item receives punctuation ranged 0 to 4, corresponding 0 to normal findings, 1 to mild (< 25% of the surface), 2 to moderate (25 – 50% of the surface), three severe (50 – 75% of the surface) and 4 to very severe (> 75% of the surface) lung involvement, respectively. The score used for the results was the mean score for each sample from both pathologists. **The evaluation of the lung injury was performed in the sections of the right upper lobe so that the atelectatic segments of the basal lung zones were not a source of overestimation of the injury (as routinely performed in studies of ARDS in animal models).**

| Experimental | **Summary of the group** | **1** | **2** | **3** | **4** | **5** | **6** | **7** | **8** | **9** | **10** |
| --- | --- | --- | --- | --- | --- | --- | --- | --- | --- | --- | --- |
| **PEEP (cm H2O)** | **12** | 9 | 12 | 12 | 10 | 9 | 11 | 9 | 12 | 12 | 12 |
| **DP (cm H2O)** | **11** | 11 | 11 | 11 | 11 | 11 | 12 | 12 | 14 | 14 | 14 |
| **PIP (cmH2O)** | **23** | 20 | 23 | 23 | 21 | 20 | 23 | 21 | 26 | 26 | 26 |
| **Vt / kg (mL / kg)** | < 6 | < 6 | < 6 | < 6 | < 6 | < 6 | < 6 | < 6 | < 6 | < 6 | < 6 |
| **RR (bpm)** | **55** | 55 | 55 | 55 | 55 | 55 | 55 | 55 | 55 | 55 | 55 |
| **pH** | **7.15** | 7.12 | 7.0 | 7.06 | 7.1 | 7.1 | 7.13 | 7.06 | 7.11 | 7.17 | 7.12 |
| **PaCO2 (mmHg)** | **92** | 91 | 105 | 102 | 102 | 60 | 93 | 89 | 67 | 65 | 68 |
| **PaO2 (mmHg)** | **137** | 227 | 85 | 96 | 108 | 180 | 190 | 150 | 137 | 140 | 120 |

**Supplementary Table E1. Ventilatory parameters received by each animal of experimental group.**

| Control | **Summary** | **1** | **2** | **3** | **4** | **5** | **6** | **7** | **8** | **9** | **10** |
| --- | --- | --- | --- | --- | --- | --- | --- | --- | --- | --- | --- |
| **PEEP (cm H2O)** | **12** | 10 | 12 | 12 | 10 | 10 | 12 | 11 | 12 | 11 | 12 |
| **DP (cm H2O)** | **14** | 14 | 14 | 14 | 14 | 14 | 14 | 14 | 14 | 14 | 14 |
| **PIP (cmH2O)** | **26** | 24 | 26 | 26 | 24 | 24 | 26 | 25 | 26 | 25 | 26 |
| **Vt / kg (mL / kg)** | **< 6** | < 6 | < 6 | < 6 | < 6 | < 6 | < 6 | < 6 | < 6 | < 6 | < 6 |
| **RR (bpm)** | **55** | 55 | 55 | 55 | 55 | 55 | 55 | 55 | 55 | 55 | 55 |
| **pH** | **7.21** | 7.17 | 7.15 | 7.16 | 7.2 | 7.13 | 7.21 | 7.23 | 7.19 | 7.2 | 7.2 |
| **PaCO2 (mmHg)** | **75** | 88 | 95 | 79 | 72 | 89 | 63 | 56 | 77 | 59 | 55 |
| **PaO2 (mmHg)** | **121** | 123 | 179 | 109 | 95 | 107 | 133 | 119 | 121 | 127 | 200 |

**Supplementary Table E2. Ventilatory parameters received by each animal of control group.**

**References.**

1. Hafner D, Germann PG: A rat model of acute respiratory distress syndrome (ARDS) Part 2, influence of lavage volume, lavage repetition, and therapeutic treatment with rSP-C surfactant. J Pharmacol Toxicol Methods 1999, 41(2-3):97-106
2. Force ADT, Ranieri VM, Rubenfeld GD, Thompson BT, Ferguson ND, Caldwell E, Fan E, Camporota L, Slutsky AS: Acute respiratory distress syndrome: the Berlin Definition. JAMA 2012, 307(23):2526-2533.
3. D. Chiumello, E. Carlesso, P. Cadringher, P. Caironi, F. Valenza, F. Polli, et al. Lung stress and strain during mechanical ventilation for acute respiratory distress syndrome. Am J Respir Crit Care Med, 178 (2008), pp. 346-355
4. Nishina K, Mikawa K, Takao Y, Shiga M, Maekawa N, Obara H. Intravenous lidocaine attenuates acute lung injury induced by hydrochloric acid aspiration in rabbits. Anesthesiology. 1998 May;88(5):1300-9.
5. Imanaka H, Shimaoka M, Matsuura N, Nishimura M, Ohta N, Kiyono H. Ventilator-induced lung injury is associated with neutrophil infiltration, macrophage activation, and TGF-beta 1 mRNA upregulation in rat lungs. Anesth Analg. 2001 Feb;92(2):428-36.
6. Belperio JA, Keane MP, Burdick MD, Londhe V, Xue YY, Li K, Phillips RJ, Strieter RM. Critical role for CXCR2 and CXCR2 ligands during the pathogenesis of ventilator-induced lung injury. J Clin Invest. 2002 Dec;110(11):1703-16.
7. Zhang W, Dai H, Lin F, Zhao C, Wang X, Zhang S, Ge W, Pei S, Pan L. Ly-6Chigh inflammatory-monocyte recruitment is regulated by p38 MAPK/MCP-1 activation and promotes ventilator-induced lung injury. Int Immunopharmacol. 2020 Jan;78:106015.
8. Li G, Liu J, Xia WF, Zhou CL, Lv LQ. Protective effects of ghrelin in ventilator-induced lung injury in rats. Int Immunopharmacol. 2017 Nov;52:85-91.

**Figure Legends.**

Figure E1. A. Tracheal cannulation with 16G Abocath. B. Configuration of the connection from the Flow I Machine to the tracheal cannula through a 3-way stopcock and a pressure sensor for Pmean.

**Figure E1**

**
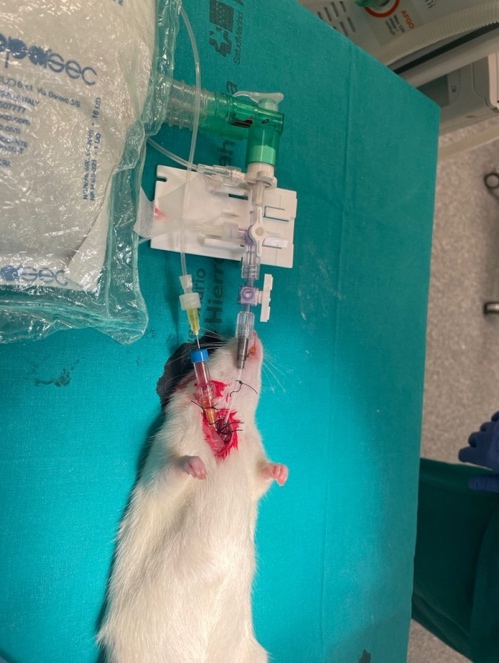

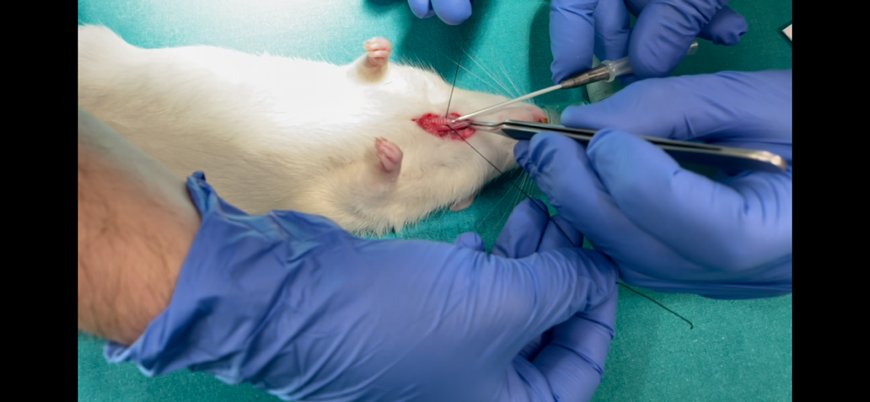
**

**Figure E2. P-V curves for each of the animals in the experimental group.**


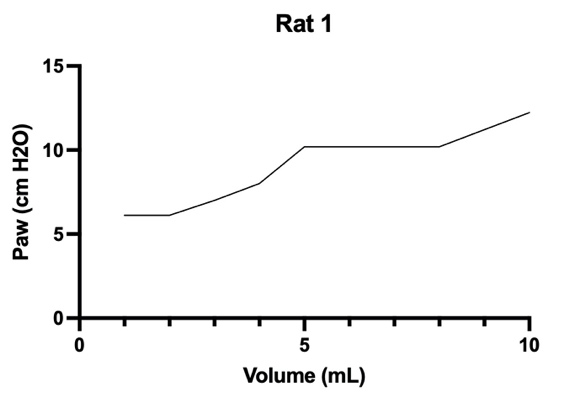

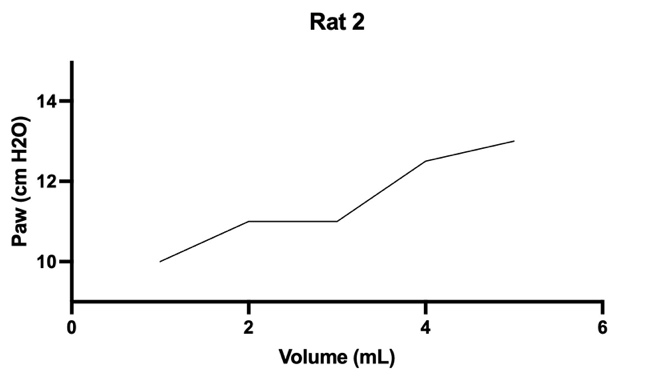

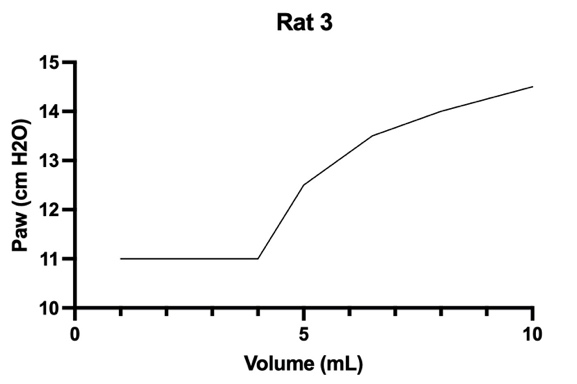

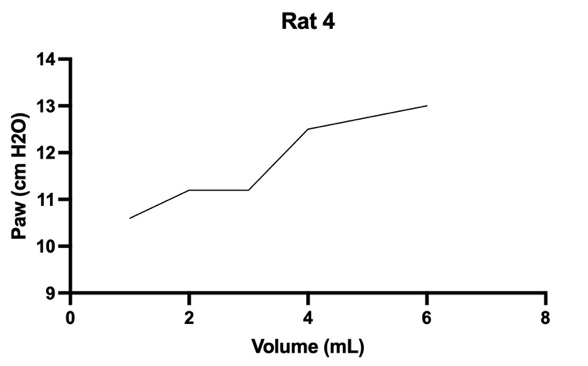

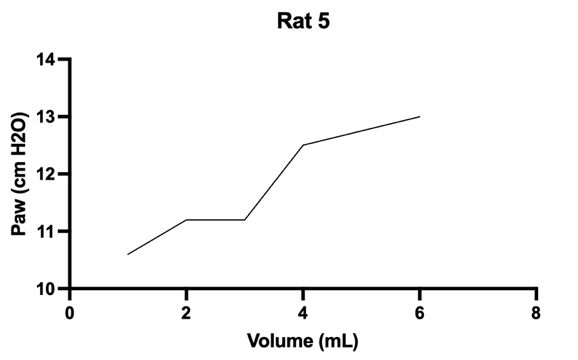

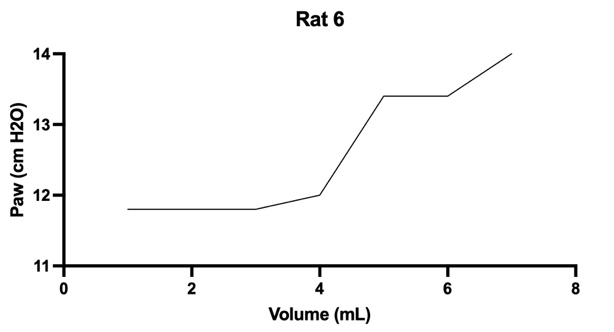

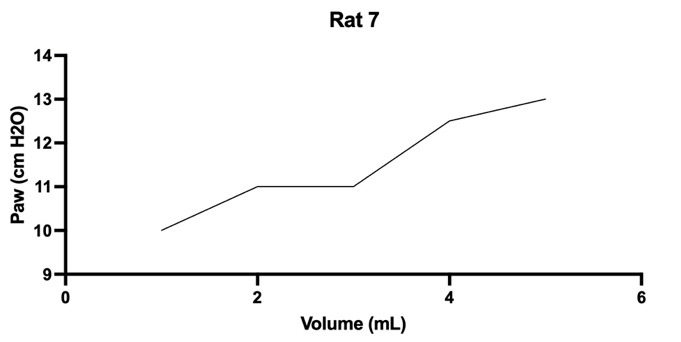

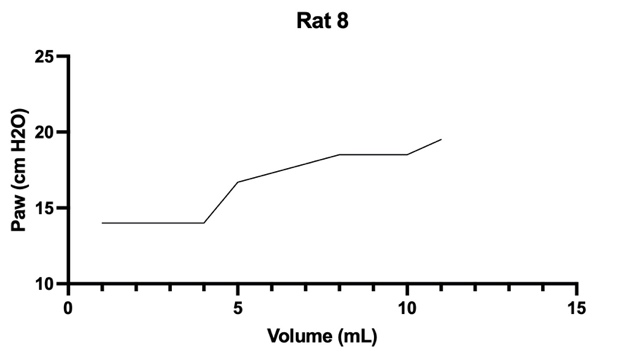

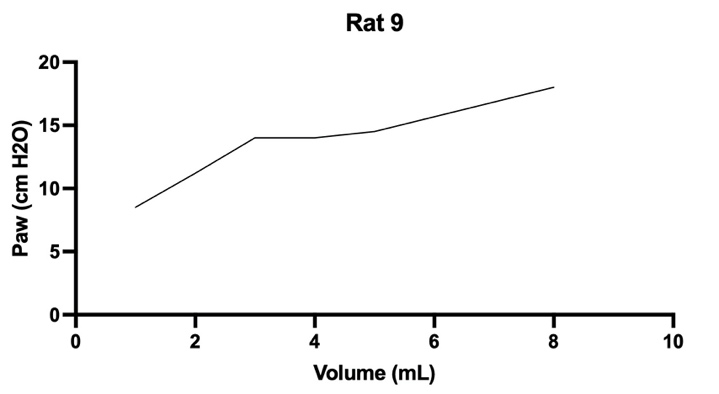

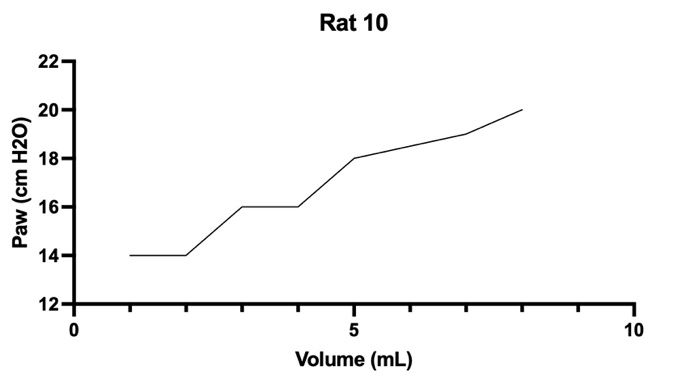

Supplement: Supplementary file 1 — Additional file 1. Details of the experimental protocol. [file 13054_2022_4184_MOESM1_ESM.docx]
